# Comparative Muscle Quality and Multiomic Analyses in Wild-Type and Yellow-Mutant *Triplophysa siluroides*: TGF-β/BMP-Mediated Covariation and Breeding for Muscle Quality

**DOI:** 10.3390/foods14244196

**Published:** 2025-12-06

**Authors:** Luyun Ni, Feiyang Li, Pengcheng Li, Yeyu Chen, Yan Liu, Jun Du, Jiansheng Lai, Ya Liu

**Affiliations:** Fisheries Research Institute, Sichuan Academy of Agricultural Sciences (Sichuan Fisheries Research Institute), Chengdu 611731, China; nluyun123@163.com (L.N.);

**Keywords:** aquaculture, muscle quality, body color variation, collagen, texture, BMP2-SMAD1-TYRP1 axis

## Abstract

This study investigated muscle quality differences between wild-type (WT) and yellow-mutant (YM) *Triplophysa siluroides*. Texture analysis showed WT *T. siluroides* had significantly greater hardness, gumminess, and resilience than YM. Histological and biochemical analyses ruled out myofiber diameter/density as drivers, instead identifying reduced collagen in YM as key, as confirmed by Picrosirius red staining, collagen quantification, and transmission electron microscopy. Transcriptomic and proteomic analyses revealed that TGF-β/BMP pathway suppression in YM resulted in downregulation of core molecules (e.g., *BMP2* and *SMAD1*), collagen-related genes (e.g., *COL1A1a* and *COL1A1b*), and ECM-related genes (e.g., *TNC* and *FN1*), potentially influencing collagen synthesis and ECM homeostasis. Notably, melanin gene *TYRP1* was also downregulated in YM *T. siluroides*, suggesting a link between pathway suppression, muscle quality alteration, and body pigmentation. The potential role of the BMP2-SMAD1-TYRP1 axis in the association between muscle quality and body colour provides novel mechanistic insights, offering molecular targets for the breeding of *T. siluroides* with superior commercial traits.

## 1. Introduction

Aquaculture, one of the world’s fastest-expanding food production sectors, is increasingly being recognized for its potential to address global food security challenges [1]. As the market demand for high-quality fish products continues to rise, aquaculture operations are focusing not only on increasing output but also on enhancing product quality [2]. Thus, improving the flesh quality of farmed fish has become a long-term goal in the aquaculture industry.

Muscle quality, particularly textural traits such as hardness, is a crucial indicator of the commercial and dietary value of teleost fish, impacting consumer acceptance and aquaculture profitability [3,4]. In fish, muscle texture is influenced primarily by muscle fiber characteristics (e.g., diameter and density) and extracellular matrix components, with collagen playing a key role in regulating muscle mechanical properties [5]. For example, increased collagen deposition enhances muscle hardness in grass carp (*Ctenopharyngodon idella*) [6], whereas reduced collagen expression is correlated with decreased muscle toughness in rainbow trout (*Oncorhynchus mykiss*) [7]. Clarifying the regulatory mechanisms of muscle texture is critical for understanding flesh quality in fish and facilitating quality improvement.

Body colour is another important phenotypic trait in fish, which is regulated by melanin synthesis and deposition. Tyrosinase-related protein 1 (TYRP1) primarily participates in the catalytic process of melanin synthesis and plays a vital role in melanogenesis by modulating the quantity and aggregation of eumelanin [8,9]. Mutations in the *TYRP1* gene are associated with various pigment deposition disorders, most notably oculocutaneous albinism type 3 (OCA3), which is characterized by reduced pigmentation in the skin, hair, and eyes [10]. These mutations are also linked to brown colouration phenotypes in horses [11], dogs [12], and fish [9].

*Triplophysa siluroides* (Cypriniformes, Nemacheilidae, *Triplophysa*), the largest nemacheilid fish endemic to China, is naturally distributed exclusively in the upper reaches of the Yellow River drainage basin [13]. As a cold-water species with irregular black-brown markings, it is highly valued in regions such as Sichuan, Gansu, and Qinghai because of its plump muscle texture, tender flesh, absence of intermuscular bones, and high nutritional value [14]. With the expansion of artificial breeding, stable yellow mutant (YM) individuals with heritable colour traits have emerged, designated YM *T. siluroides* in this study. These mutants have gained increasing market favour and greater economic value due to their rarity and ornamental appeal, showing significant potential for development and application [15]. They not only serve as a natural model for exploring phenotypic variation but also have implications for aquaculture breeding. Since body colour and muscle quality are both core traits involved in variety improvement, clarifying their correlation and regulatory mechanisms can provide a basis for the directional breeding of *T. siluroides* with superior commercial characteristics.

Notably, emerging evidence suggests potential covariation between body colour and muscle quality in some fish species. For example, yellow-mutant and wild-type northern snakehead (*Channa argus*) exhibited differences in muscle texture and metabolic profiles, suggesting an uncharacterized link between these traits [16]. In *T. siluroides*, we previously conducted preliminary studies examining differences in body colouration and muscle quality. Comparative analysis of the nutritional composition of muscles from wild-type (WT) and YM *T. siluroides* revealed potential variations in muscle fiber density and diameter, and revealed that compared with WT *T. siluroides*, YM *T. siluroides* presented significantly higher levels of total amino acids, essential amino acids, and delicious amino acids [15]. Furthermore, a previous transcriptomic study in *T. siluroides* identified candidate genes associated with skin colour variation, providing a foundation for exploring colour-related regulatory mechanisms [17]. However, the mechanistic link between body colour variation and muscle quality remains unclear, currently limiting the application of *T. siluroides* in selective breeding programmes.

The TGF-β/BMP (transforming growth factor beta/bone morphogenetic protein) signaling pathway is a conserved regulatory axis in vertebrates, and its dual functions provide clues for interpreting the above-mentioned association: it modulated collagen synthesis and ECM (extracellular matrix) homeostasis in muscle via SMAD (suppressor of mother against decapentaplegic)-dependent transcription of collagen genes (e.g., *COL1A1*, *COL1A2*) [18,19] and regulates melanogenesis by promoting tyrosinase expression and melanocyte regeneration [20,21]. This dual functionality makes it a plausible candidate for coordinating body colour and muscle quality, yet its role in *T. siluroides* remains unexamined.

This study aims to compare muscle texture traits and underlying structural factors (myofiber morphology, collagen content) between WT and YM *T. siluroides*, identify key molecular pathways regulating these differences through transcriptomic and proteomic analyses, and explore whether these pathways simultaneously modulate body color and muscle quality, revealing a mechanism for trait covariation. This work is expected to clarify the basis of muscle quality differences in *T. siluroides* and provide theoretical support for the selective breeding of this species, facilitating the development of new varieties with both desirable color and superior muscle quality.

## 2. Materials and Methods

### 2.1. Fish Preparation

The YM *T. siluroides* were derived from selective breeding of natural yellow mutants combined with artificial propagation. Briefly, wild individuals with stable yellow body color were selected as founding parents for artificial insemination. F_1_ and F_2_ generations were reared under standardized environmental conditions, and F_2_ offspring with a genetically stable yellow phenotype were used as experimental samples. Artificially cultured WT and YM *T. siluroides*, sourced from a breeding company in Sichuan, presented average weights of 195.95 g ± 3.86 g and 196.23 g ± 8.57 g, respectively. Following a one-week acclimation period at 16 ± 0.5 °C in the laboratory, during which they were fed commercial feed twice daily, all fish were confirmed to be in good health and free of disease.

### 2.2. Texture Determination

Texture profile analysis (TPA) was performed on the muscle tissue of WT and YM *T. siluroides* using a TA-XTplusC texture analyzer (Stable Micro Systems, Godalming, Surrey, United Kingdom) to evaluate various parameters, including hardness, chewiness, springiness, cohesiveness, gumminess, and resilience. Dorsal muscle samples (*n* = 6) that were 2 × 2 × 1 cm in size were tested with a P/6R radius aluminum probe at a test speed of 5 mm/s, a pretest speed of 2 mm/s, and a post-test speed of 5 mm/s. The compression was applied twice with a 5-s interval at a force of 5 g and strain rate of 50% under controlled temperature conditions ranging from 20 to 25 °C.

### 2.3. Histological Analysis

#### 2.3.1. H&E Staining

Dorsal muscle samples (*n* = 3) from WT and YM *T. siluroides* were sampled and then fixed in 4% paraformaldehyde (PFA) solution at room temperature for 24 h. Following post-fixation, the samples were dehydrated in an ethanol gradient, embedded in paraffin, and sectioned into 5-μm-thick slices, followed by staining with hematoxylin and eosin (H&E). The sections were mounted with neutral resin, dried, and examined using an optical microscope (DM500; Leica, Wetzlar, Germany) for imaging. Whole-slide imaging was performed using a Leica microsystem (DM1000; Leica, Germany). Six random fields were selected for photography. Muscle fiber diameters were analysed using Image-Pro Plus 6.0 software. The quantity of muscle fibers in each field was counted and standardized to the number of muscle fibers per square millimeter.

#### 2.3.2. Picrosirius Red Staining

Collagen fibers in the dorsal muscles of WT and YM *T. siluroides* (*n* = 3) were stained using the Picrosirius red method. Fresh muscle tissues were fixed in 4% PFA, dehydrated in an alcohol gradient, cleared in xylene, embedded in paraffin, and sectioned into 5 µm slices. The sections were deparaffinized, stained with Picrosirius red solution, cleared in xylene, mounted with neutral resin, and examined for positive signals under a light microscope (DM500; Leica, Germany). Whole-slide imaging was conducted using a Leica Microsystems system (DM1000, Leica, Germany).

Collagen fibers in muscle tissues were stained red, and the collagen fiber-positive signals were quantified in the dorsal muscles of WT and YM *T. siluroides*. Each sample was captured at 100× magnification, and the average optical density (AOD) of positive expression per unit area in three fields of view was determined using Image-Pro Plus 6.0 (AOD = total optical density/total area of view). Six samples from each colour morph of *T. siluroides* were analysed.

#### 2.3.3. Transmission Electron Microscopy (TEM) Analysis

Muscle tissues from WT and YM *T. siluroides* (*n* = 3) were fixed overnight at 4 °C in 2.5% glutaraldehyde, followed by a 2 h fixation at room temperature in 1% osmium tetroxide. Subsequent steps included dehydration in acetone gradients, infiltration and embedding in Epox 812 resin (Beijing Zhongjing Keyi Technology Co., Ltd. Beijing, China). Ultrathin sections of 60–90 nm were cut using a diamond knife, mounted on metal grids, and stained with uranyl acetate and lead citrate. Observation and imaging were performed using a JEM-1400-FLASH transmission electron microscope (JEOL Ltd., Tokyo, Japan). The percentage of collagen fibers relative to the total area of the observed region (%) was quantified using Image J (fiji).

### 2.4. Quantification of the Collagen Content in Muscle Tissue

In accordance with the methodology established by Hou et al. [22] the Total Collagen Assay Kit (GMS50326.2, GenMed Scientifics Inc., Boston, MA, USA) was utilized to assess the total collagen content in the dorsal muscles of WT and YM *T. siluroides*. Following the kit instructions, a standard curve was established, and the collagen content of the muscle tissue was measured after grinding. Six different samples were analysed per group (*n* = 6).

### 2.5. Transcriptome Sequencing and Analysis

#### 2.5.1. Total RNA Extraction and Transcriptome Sequencing

Transcriptome sequencing was conducted on muscle tissues from WT and YM *T. siluroides*. Total RNA was extracted from muscle samples (*n* = 3) using TRIzol reagent (Invitrogen, Carlsbad, CA, USA) following the manufacturer’s protocol. RNA quality (degradation, purity, and integrity) was assessed by 1% agarose gel electrophoresis and an Agilent Bioanalyzer 2100 system (Agilent Technologies; Palo Alto, CA, USA). Eukaryotic mRNA was enriched using oligo (dT) magnetic beads, whereas prokaryotic mRNA was enriched by rRNA depletion with the Ribo-Zero™ Magnetic Kit (Epicentre, Madison, WI, USA). The enriched mRNA was fragmented and converted to cDNA using random primers. Second-strand cDNA synthesis was performed with DNA polymerase I, RNase H, and dNTPs. After end-repair, the cDNA fragments were ligated to Illumina sequencing adapters. After purification and amplification, 150 bp paired-end sequencing was performed on the Illumina NovaSeq 6000 platform (Illumina, San Diego, CA, USA) by Gene Denovo Biotechnology Co., Ltd. (Guangzhou, China).

#### 2.5.2. Transcriptome Data Analysis

After sequencing, the raw data underwent filtering to remove adapter sequences, reads containing over 10% unknown nucleotides, and low-quality reads (q-value ≤ 20) using fastp to obtain clean reads [23]. These clean reads were aligned to the reference genome of *T. siluroides* using HISAT2 software (v2.2.1) [24]. Subsequently, transcript reconstruction was performed based on the HISAT2 alignment results using Stringtie (v3.0) [25], and the gene expression levels in each sample were computed using RSEM (v1.3.3) [26]. Unigenes were functionally annotated by comparing them against the Swiss-Prot database (http://www.expasy.ch/sprot, accessed on 6 May 2025), NCBI nonredundant (NR) protein database (http://www.ncbi.nlm.nih.gov, accessed on 6 May 2025), Kyoto Encyclopedia of Genes and Genomes (KEGG) database (http://www.genome.jp/kegg, accessed on 6 May 2025), and COG/KOG database (http://www.ncbi.nlm.nih.gov/COG, accessed on 6 May 2025) using the BLASTx program (v2.16.0) (http://www.ncbi.nlm.nih.gov/BLAST, accessed on 6 May 2025) (E-value threshold of 1 × 10^−5^). To assess expression level differences between WT and YM *T. siluroides*, gene expression levels were quantified using TPM values (transcripts per kilobase of exon model per million mapped reads) with RSEM and DESeq2 [26,27]. Differentially expressed genes (DEGs) were identified based on |log_2_FC| > 1 and a false discovery rate (FDR) < 0.05. Subsequently, the DEGs underwent Gene Ontology (GO) functional enrichment and KEGG enrichment analyses using the clusterProfiler package (v4.14.6) in R, with the results visualized through the ggplot2 package (v4.0.0) [28].

#### 2.5.3. Gene Set Enrichment Analysis of DEGs

Gene set enrichment analysis (GSEA) was conducted to compare specific gene sets within pathways between WT and YM *T. siluroides*. GSEA could address the limitations of traditional enrichment analysis in capturing information from low-effect genes. GSEA software and MSigDB were utilized to identify differentially enriched GO terms and pathways between the two groups [29]. Gene expression data was input, genes were ranked using the Signal2Noise method, and enrichment scores (ES), *p*-values, and FDR values were calculated using default parameters. Normalized enrichment scores (|NES|) were obtained, where pathways with |NES| > 1, NOM *p*-value < 0.05, and FDR q-value < 0.25 were considered statistically significant pathways. Larger absolute values of NES corresponded to smaller FDR values, indicating greater reliability of the analysis results. Additionally, an assessment of collagen-related gene expression levels was conducted, and the results were visualized as a heatmap.

### 2.6. Proteome Sequencing and Analysis

#### 2.6.1. Total Protein Extraction and DIA (Data-Independent Acquisition) Data Collection

Total protein was extracted from the muscle tissues of WT and YM *T. siluroides* (*n* = 3) via homogenization by placing the tissues in a lysis buffer containing 1% SDS, 8 M urea, and 1 mg/mL protease inhibitor cocktail. The mixture was homogenized using a high-throughput tissue homogenizer and then centrifuged at 14,000× *g* for 20 min at 4 °C to obtain the supernatant. The protein concentration was determined using the Bicinchoninic Acid (BCA) method. The protein sample preparation process included denaturation, reduction, alkylation, tryptic digestion, and peptide cleanup using the iST Sample Preparation Kit (PreOmics, Gauting, Germany) according to the manufacturer’s instructions. Initially, 50 μL of Lyse buffer was heated at 95 °C for 10 min at 1000 rpm. After cooling, trypsin digestion buffer was added, and the sample was incubated at 37 °C for 2 h at 500 rpm. The digestion was stopped with a stop buffer, followed by sample cleanup, desalting, and peptide elution with elution buffer. Finally, the peptides were lyophilized using a SpeedVac (Thermo Fisher Scientific, Waltham, MA, USA). Following desalination and lyophilization, the peptides were reconstituted in a 0.1% formic acid aqueous solution and subjected to LC–MS/MS analysis. The UltiMate 3000 liquid chromatography system (Thermo Fisher Scientific, Waltham, MA, USA) was connected to the timsTOF Pro2 Mass spectrometer by Bruker Daltonics (Billerica, MA, USA). Data were acquired using data-independent acquisition (DIA) in the diaPASEF mode.

#### 2.6.2. Spectrometric Data Analysis

Data from the DIA were analysed using Spectronaut 18 (Biognosys AG, Schlieren, Switzerland) with standard settings. Retention time prediction was performed with the dynamic iRT setting. Spectronaut was applied to perform data extraction with mass calibration and adjusted the extraction window dynamically based on iRT calibration and gradient stability [30]. A 1% q-value (FDR) threshold was applied at both the precursor and protein levels. Decoys were generated with a mutated configuration involving random amino acid position swaps (min = 2, max = length/2). Local normalization was used for data normalization. The major group quantities were calculated using the average of the top 3 filtered peptides that met the 1% q-value cut-off. Protein functions and classifications were determined through searches against the GO, KEGG, and COG/KOG databases. Significantly differentially expressed proteins (DEPs) were identified based on protein quantification, with *p* values < 0.05 and fold change (FC) > 1.2 indicating statistical significance. GO and KEGG functional enrichment analyses were subsequently conducted for the DEPs.

#### 2.6.3. Integrated Analysis of the Transcriptome and Proteome

The quantitative relationships between genes and proteins were investigated by correlation analysis using R software (version 3.5.1). Following this analysis, a nine-quadrant map was devised to elucidate the interplay of gene expression changes across the transcriptomic and proteomic levels.

### 2.7. Quantitative Real-Time PCR (qRT-PCR) Analysis

A total of 12 genes were chosen for validation of RNA-seq results through quantitative real-time PCR (qRT-PCR). Total RNA was extracted from muscle tissues of WT and YM *T. siluroides*, followed by cDNA synthesis. The primers used for the target genes are listed in Table A1. Each assay was conducted in triplicate for every sample using the ABI 7500 FAST real-time PCR system (Applied Biosystems, Waltham, MA, USA) and 2x SG Fast qPCR Master Mix (Sangon, Shanghai, China). For qRT-PCR, reactions were subjected to an initial denaturation at 95 °C (3 min), then 45 cycles of 95 °C for 15 s (denaturation), 60 °C for 30 s (annealing), and 72 °C for 30 s (extension). The expression stability of the reference genes *β-actin*, glyceraldehyde-3-phosphate dehydrogenase (*GAPDH*), and elongation factor 1-alpha (*EF1a*) was evaluated using the NormFinder program, in accordance with Minimum information for publication of quantitative real-time PCR experiments (MIQE) guidelines [31]. The relative expression of target genes in hybrid sturgeons was subsequently quantified using the 2^−ΔΔCt^ method [32].

### 2.8. Statistical Analysis

The statistical analysis was performed using R project (version 4.3.3) and involved analysis of variance, followed by independent t tests for the measured data. The data are displayed as mean ± standard error, with statistical significance denoted by *p* < 0.05. A bar plot for qRT-PCR validation was generated using GraphPad Prism 9. A schematic diagram of molecular mechanisms was drawn using Adobe Illustrator CS6 (V16.0).

## 3. Results

### 3.1. Muscle Textural Characteristics of WT and YM T. siluroides

The texture characteristics of the dorsal muscles were compared between WT and YM *T. siluroides*. As shown in Table 1, the hardness, gumminess, and resilience of WT *T. siluroides* muscles were significantly greater than those of their YM counterparts (*p* < 0.05). Additionally, WT *T. siluroides* presented slightly greater chewiness, springiness, and gumminess of muscles than did YM *T. siluroides*, although these differences were not statistically significant.

### 3.2. Muscle Fiber Structure of WT and YM T. siluroides

**Figure 1:**
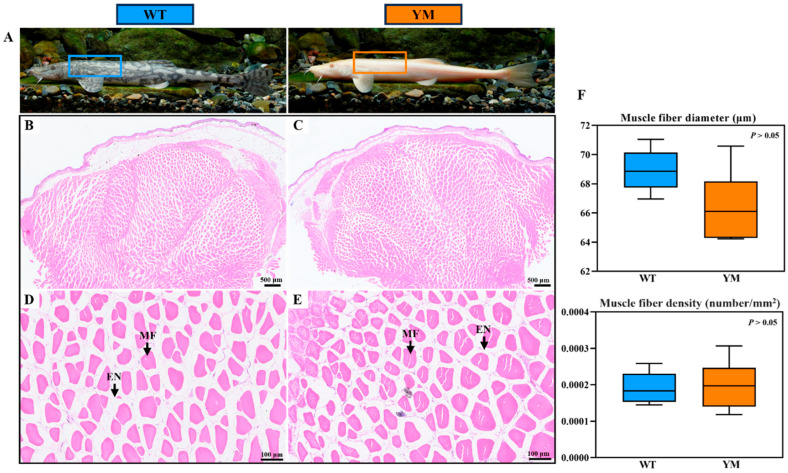
Histological features of muscle fiber in WT and YM *T. siluroides*. (**A**): The sampling sites in the muscles of WT and YM *T. siluroides*; (**B**,**D**): The microstructure of WT *T. siluroides* muscle; (**C**,**E**): The microstructure of YM *T. siluroides* muscle. (**F**): Comparison of muscle fiber diameter and density between WT and YM *T. siluroides*. En. Endomysium; MF. Muscle fiber.

Muscle tissue samples from the same location from WT and YM *T. Siluroides* were obtained and subjected to H&E staining for histological analysis, as shown in Figure 1A. The muscle predominantly consisted of round and polygonal fibers that were neatly arranged and separated by thin endomysium (Figure 1B–E). There were no significant differences in muscle fiber diameter or density between WT and YM *T. siluroides* (*p* > 0.05; Figure 1F).

### 3.3. Muscle Collagen Content of WT and YM T. siluroides

**Figure 2:**
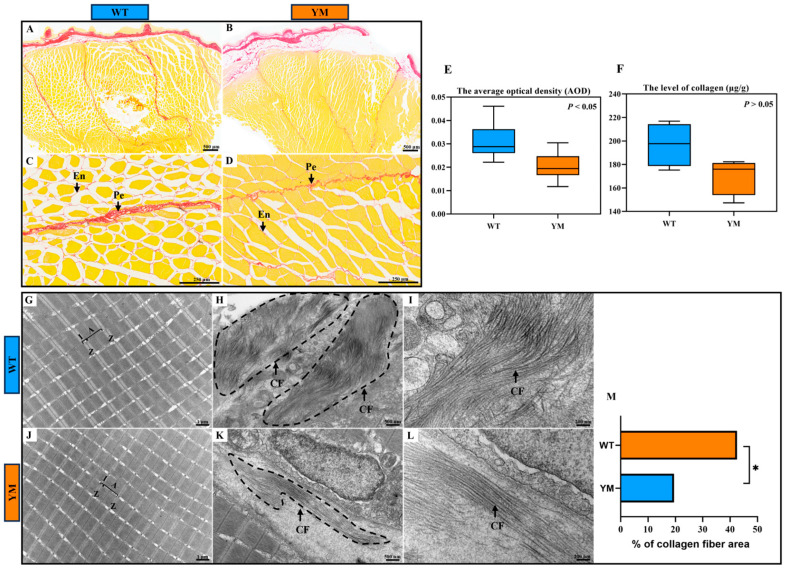
Muscle collagen content investigation in the dorsal muscles of WT and YM *T. siluroides*. (**A**–**D**): Picrosirius Red staining of the muscle samples. (**E**): Collagen area quantification based on the stained regions. (**F**): Tissue collagen content quantified by colorimetric assay. (**G**–**L**): T TEM analysis of the dorsal muscles in WT and YM *T. siluroides* revealed significant differences in collagen fiber abundance (dashed line box). (**M**): Quantitative analysis of the percentage of collagen fiber area in muscle tissues from WT and YM *T. siluroides*. * denotes a significant difference at *p* < 0.05. A. A band; CF. Collagen fiber; En. Endomysium; I. I band; Pe. Perimysium; Z. Z line.

The collagen content in the dorsal muscles of WT and YM *T. siluroides* was evaluated using Picrosirius Red staining. The muscle fibers were demarcated by the endomysium, with the thicker perimysium dividing them into bundles (Figure 2A–D). Collagen was predominantly localized in the endomysium and perimysium connective tissues, and appeared red in colour. Image analysis software was used for quantification of the collagen area, with the average optical density (AOD) representing the mean optical density of pixels within the collagen fiber distribution area. This approach aims to standardize staining variations and measure collagen levels precisely. A higher AOD value indicates a greater density of collagen fibers, whereas a lower value suggests a lower collagen content. As shown in Figure 2E, the AOD value of Picrosirius Red staining in the dorsal muscles of WT *T. siluroides* was significantly greater than that of YM *T. siluroides* (*p* < 0.05).

To accurately quantify the collagen content in the dorsal muscles of WT and YM *T. siluroides*, a colorimetric method was employed for quantitative analysis. The results revealed that the collagen content in the muscle of WT *T. siluroides* was greater than that in the muscle of YM *T. siluroides*, although this difference was not statistically significant (Figure 2F; *p* > 0.05).

Additionally, transmission electron microscopy (TEM) was utilized to examine the dorsal muscles of WT and YM *T. siluroides*. The myofibrillar structures showed no distinct differences between the two groups: in both WT and YM individuals, the myofibrils were neatly arranged with well-defined sarcomere architectures. The A bands (dark bands) and I bands (light bands) were clearly delineated, whereas the Z lines appeared as continuous, straight dense lines that evenly divided the sarcomeres (Figure 2G,J). The myofilaments within the sarcomeres were densely packed without discernible dissociation, and the boundaries between adjacent myofibrils remained distinct. Notably, significant differences were observed in the distribution of collagen fibers between the two groups. In WT *T. siluroides*, collagen fibers exhibited a notably higher quantity and denser distribution, forming a compact network around the myofibrils. In contrast, the collagen fibers in YM *T. siluroides* were sparser, with a more scattered arrangement (Figure 2H,I,K,L). The quantification of collagen fiber percentage relative to the total area of the observed region in the muscle tissues of WT and YM *T. siluroides* indicated a significantly higher value in WT *T. siluroides* compared to YM *T. siluroides* (Figure 2M).

### 3.4. Differential Transcriptomic and Proteomic Analyses

**Figure 3:**
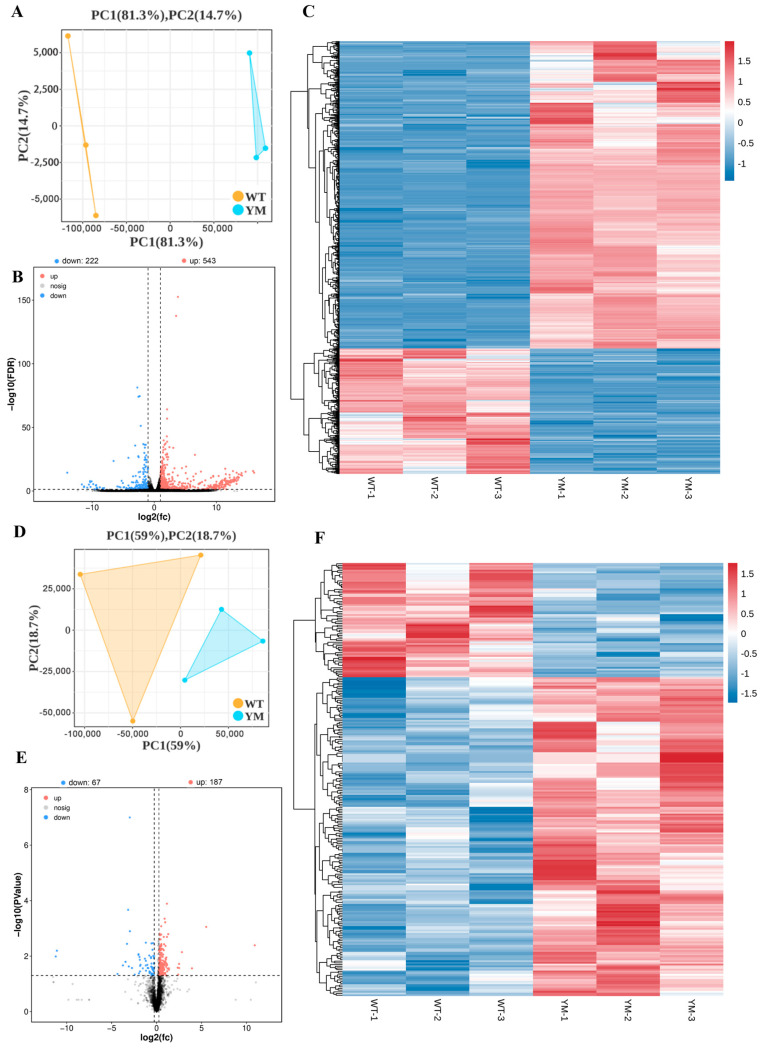
Transcriptomic and proteomic profiling of WT and YM *T. siluroides* muscle. (**A**): PCA plot of muscle samples for transcriptome. (**B**): Volcano plot of DEGs between WT and YM *T. siluroides*. The blue dots represent down-regulation of gene expression, the red dots indicate upregulation of gene expression, and the black dots signify genes with no significant differences in expression. (**C**): The heatmap of DEGs between WT and YM *T. siluroides*. (**D**): PCA plot of muscle samples for proteome. (**E**): Volcano plot of DEPs between WT and YM *T. siluroides*. (**F**): The heatmap of DEPs between WT and YM *T. siluroides*.

Transcriptomic and proteomic sequencing were performed on muscle tissues from WT and YM *T. siluroides*. Transcriptome sequencing of six muscle-derived cDNA libraries generated a total of 35,954,239,200 raw reads, with quality scores of Q20 > 99.2% and Q30 > 97.8% (Table 2). The PCA results depicted in Figure 3A show distinct clustering of WT and YM muscle samples. The volcano plots in Figure 3B show 426 upregulated and 189 downregulated DEGs (FDR < 0.05, |log_2_FC| > 1). The heatmap in Figure 3C, normalized using z scores, illustrates gene expression levels and hierarchical sample clusters, revealing separate clusters for upregulated and downregulated genes. The proteomic analysis, as shown in Figure 3D, confirmed good reproducibility among the samples. The proteomic analysis presented in Figure 3E revealed 153 upregulated and 78 downregulated DEPs (*p* < 0.05, FC > 1.2). Consistent with the transcriptomic data, clear clustering of upregulated and downregulated proteins is evident in Figure 3E.

**Figure 4:**
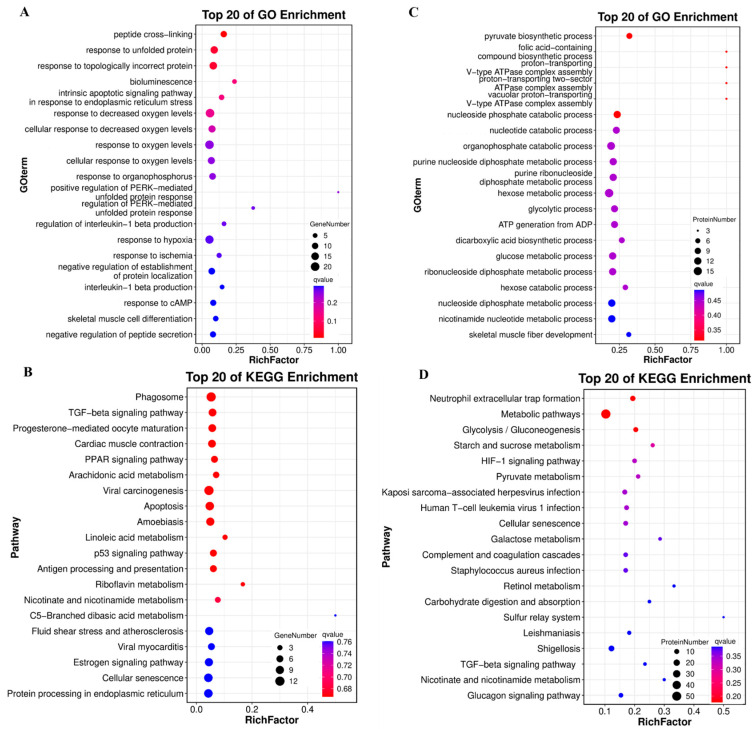
GO and KEGG enrichment of DEGs and DEPs in WT and YM *T. siluroides* muscle. (**A**): Bubble plot of the top 20 GO biological process (BP) terms enriched in DEGs. (**B**): Bubble plot of the top 20 KEGG enriched pathways for DEGs. (**C**): Bubble plot of the top 20 GO BP terms enriched in DEPs. (**D**): Bubble plot of the top 20 KEGG enriched pathways for DEPs. RichFactor: Ratio of DEGs/DEPs enriched in a term/pathway to total annotated unigenes/proteins, indicating enrichment degree.

GO and KEGG enrichment analyses were performed on DEGs (transcriptome) and DEPs (proteome). For DEGs, the most enriched terms prioritized peptide cross-linking, the hypoxic response, and apoptosis-related processes (Figure 4A). KEGG pathway analysis highlighted the phagosome, TGF-β signaling, and muscle contraction pathways (Figure 4B). For DEPs, the top 20 GO terms focused on metabolic processes (e.g., pyruvate biosynthesis, glycolysis; upper right), whereas the KEGG pathways were enriched for glycolysis, HIF-1 signaling, and metabolic networks (Figure 4C,D). Additionally, we also observed the enrichment of DEPs in the TGF-β signaling pathway.

### 3.5. GSEA and Multiomic Co-Expression Analysis

**Figure 5:**
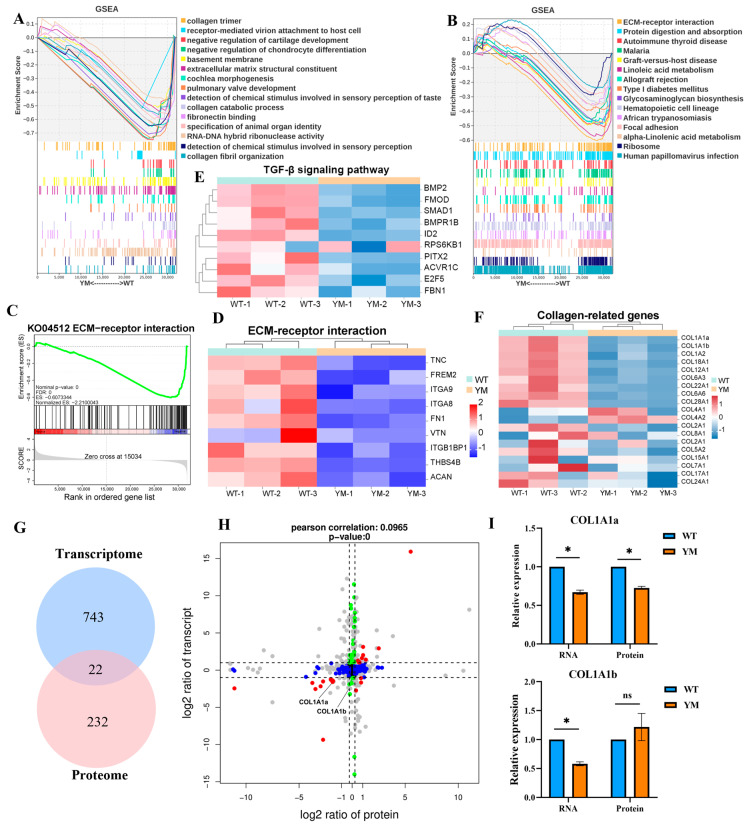
Multi-omics co-expression analysis of collagen and ECM pathways in WT and YM *T. siluroides* muscle. (**A**): GSEA plot of GO terms, revealing suppression of relevant biological processes in YM *T. siluroides* muscle. (**B**): GSEA plot of KEGG pathways, indicating downregulation of associated signaling pathways in YM. (**C**): GSEA curve for ECM-receptor interaction (KEGG: ko04512). (**D**): Heatmap of some ECM-receptor interaction pathway genes. (**E**): Heatmap of genes of TGF-βsignaling pathway. (**F**): Heatmap of collagen-related genes. (**G**): Integrated analysis of transcriptome and proteome, Venn diagram of DEGs (*n* = 743) and DEPs (*n* = 232), with 22 overlaps. (**H**): Nine-quadrant plot of the integrated analysis of transcriptome and proteome. (**I**): Relative expression levels of *COL1A1a* and *COL1A1b* at RNA and protein levels in WT and YM *T. siluroides* muscle. * = *p* < 0.05; ns = non-significant.

GSEA of the transcriptomic data revealed significant suppression of collagen-related GO terms, such as collagen trimer, extracellular matrix structural constituent, collagen catabolic process and collagen fibril organization, as well as the KEGG ECM-receptor interaction pathway in YM *T. siluroides* muscle (Figure 5A,B). The GSEA curve for ECM-receptor interaction pathway (KO04512) revealed pathway suppression (Figure 5C), with *TNC* (Tenascin C), *FREM2* (FRAS1 related extracellular matrix 2), and *ITGA8* (Integrin subunit alpha 8) showing downregulated expression in YM *T. siluroides* (Figure 5D). Given the enrichment of collagen-related terms, the TGF-β signaling pathway, and the ECM-receptor interaction signaling pathway, we further analysed key genes associated with these terms or pathways. Specifically, we examined the gene expression levels of certain genes enriched in the TGF-β signaling pathway, which revealed significant downregulation of the *BMP2*, *FMOD*, and *SMAD1* genes in the muscle of YM *T. siluroides* (Figure 5E). The heatmap depicting collagen-related genes illustrated widespread downregulation in YM *T. siluroides*, especially in the *COL1A1* subtypes (*COL1A1a*, *COL1A1b*; Figure 5F). The correlation between the transcriptomic and proteomic data revealed 22 overlapping DEGs and DEPs among the 743 transcriptomic DEGs and 232 proteomic DEPs (Figure 5G). A 9-quadrant plot (*x*-axis: log_2_ (transcript fold change); *y*-axis: log_2_ (protein fold change); Pearson r = 0.0965) was used to visualize expression consistency, with red dots representing *COL1A1* subtypes. Among these genes, *COL1A1a* showed significant and consistent downregulation in YM, while *COL1A1b* exhibited a non-significant change at RNA level but significant reduction at the protein level (Figure 5H,I).

### 3.6. Potential Molecular Mechanisms Underlying Differences in Muscle Hardness

**Figure 6:**
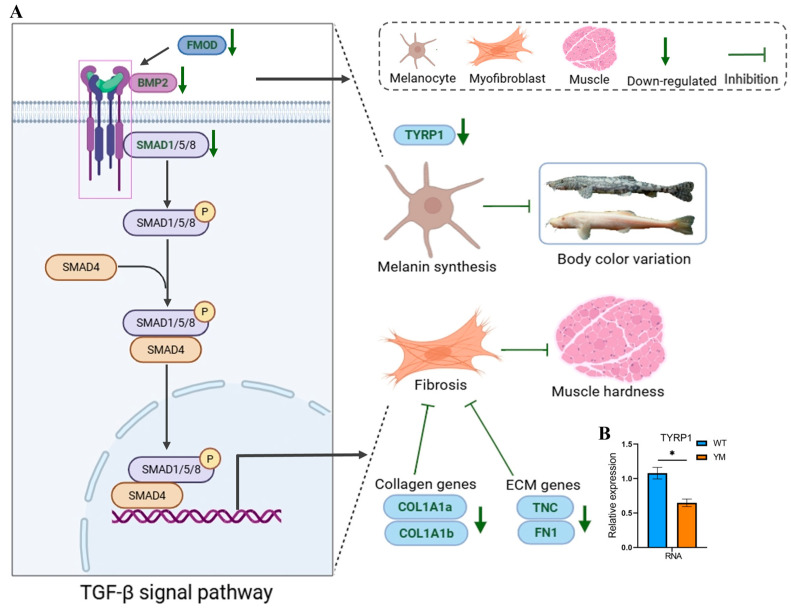
Potential molecular mechanisms underlying differences in muscle hardness between WT and YM *T. siluroides*. (**A**): Potential molecular networks. P denotes phosphorylation. (**B**): Bar plot of *TYRP1* RNA expression. * denotes a significant difference at *p* < 0.05.

To investigate the underlying mechanism, a potential regulatory molecular network was analysed (Figure 6). Integrated transcriptomic, proteomic, and molecular biological analyses revealed that the TGF-β/BMP signaling axis was responsible for the phenotypic differences in *T. siluroides*. In the muscle of YM *T. siluroides*, the key pathway molecules *BMP2*, *FMOD*, and *SMAD1* were downregulated, leading to the suppression of collagen (*COL1A1a*, *COL1A1b*) and ECM-related genes (*TNC*, *FN1*). This inhibition disrupted collagen synthesis and ECM stability, which may result in reduced muscle hardness. Moreover, the attenuation of the TGF-β/BMP pathway in YM *T. siluroides* was associated with a significant decrease in *TYRP1* (a regulator of melanin synthesis) RNA expression (Figure 6B, *p* < 0.05), influencing body color variation. These findings suggest a potential connection between body colour divergence, reduced muscle hardness, and collagen depletion mediated by the TGF-β/BMP pathway.

### 3.7. qRT-PCR Verification

**Figure 7:**
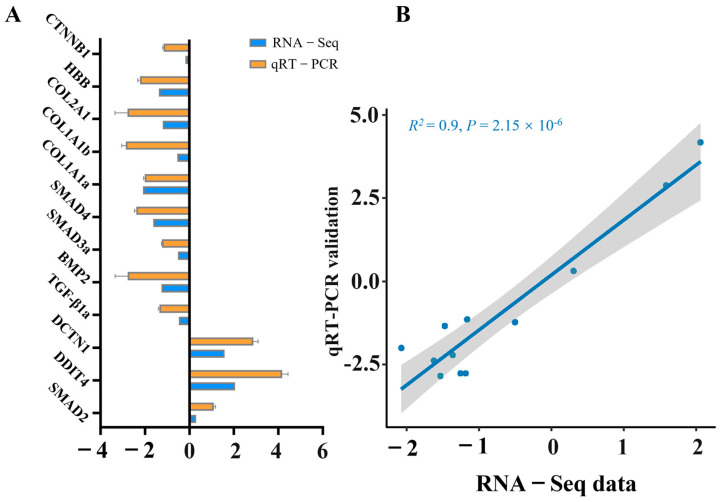
Validation of RNA-seq data through qRT-PCR analysis of selected genes. (**A**) Comparative analysis between RNA-seq and qRT-PCR outcomes. qRT-PCR data are presented as means ± SE (*n* = 3). (**B**) Scatter plot depicting the correlation coefficient between qRT-PCR and RNA-seq results.

To validate the RNA sequencing results, 12 genes were subjected to confirmation via qRT-PCR. In accordance with the MIQE guidelines, the NormFinder program was used to evaluate the expression stability of three candidate reference genes: *β-actin*, *GAPDH*, and *EF1a*. The calculated stability values for *β-actin*, *GAPDH*, and *EF1α* were 0.21, 0.28, and 0.56, respectively. Given its superior stability, *β-actin* was selected as the reference gene for data normalization in this study. The resulting fold changes derived from qRT-PCR were subsequently compared with the RNA-seq expression data. As depicted in Figure 7, all the genes presented comparable expression patterns in both the qRT-PCR and RNA-seq analyses. A significant correlation was identified between the qRT-PCR and RNA-seq results, confirming the alignment with the results of the transcription analysis.

## 4. Discussion

### 4.1. Confirmation of Muscle Hardness Differences and Irrelevance of Myofiber Traits

TPA, a widely accepted method for assessing muscle textural properties, provides quantitative data on parameters such as hardness, chewiness, and resilience [33,34]. In the present study, TPA demonstrated that WT *T. siluroides* presented significantly greater muscle hardness than did YM individuals, with consistent trends observed in gumminess and resilience, although not all parameters reached statistical significance. This finding confirmed the divergence in muscle texture between the WT and YM *T. siluroides*. The sensory perception of hardness refers to the peak force needed to compress a food item between the molars, serving as a vital determinant of muscle quality that significantly influences consumer preference and culinary appropriateness [35,36]. Furthermore, our previous study, which involved a comparative analysis of the nutritional composition of muscles from WT and YM *T. siluroides*, revealed that compared with WT *T. siluroides*, YM *T. siluroides* presented significantly higher levels of total amino acids, essential amino acids, and delicious amino acids, indicating superior nutritional characteristics [15].To date, numerous studies have examined the mechanisms of colour variation in fish, comparing the nutritional composition of muscle or differences in muscle quality among fish of varying colours [16,17]. Nevertheless, there has been a lack of research investigating the underlying mechanisms responsible for variations in muscle quality among fish of different colours. Our results lay the foundation for further investigations into the mechanistic basis of muscle quality differences in *T. siluroides*.

Essentially, the texture of flesh is predominantly dictated by two factors: muscle fibers and collagen content [37,38]. Myofibers, the primary structural components of muscle tissue, have been linked to textural variations among different fish species. Previous studies have shown a negative relationship between muscle fiber density and diameter, whereas a positive association exists between the density and hardness and chewiness of muscle tissue [39,40]. Additionally, the collagen content in fish flesh is positively correlated with muscle hardness [6]. Feeding grass carp with broad beans significantly increases muscle hardness, resulting in a condition known as “Crispy Grass Carp”, which is characterized by a significant decrease in muscle fiber diameter and a notable increase in muscle fiber density [41]. In the present study, histological analysis via H&E staining revealed no significant differences in myofiber diameter or density between WT and YM *T. siluroides*. This result excludes myofiber-related traits as the primary drivers of the observed hardness difference, prompting a focus on alternative factors—specifically, collagen content, which is a well-documented regulator of tissue mechanical properties.

### 4.2. Reduced Collagen Content Underlies Differences in Muscle Hardness

Collagen, a crucial extracellular matrix protein, is closely associated with muscle firmness [33]. Its cross-linked structure tightly binds fibers, weaving them into a stable network with improved toughness and mechanical strength [5,42]. For example, grass carp (*Ctenopharyngodon idellus*) fed a broad bean diet exhibit increased transcription of type I collagen, resulting in increased collagen content and muscle hardness [43]. Similarly, yellow croaker (*Larimichthys crocea*) receiving hydroxyproline-supplemented feed shows a higher collagen content and increased muscle hardness [44]. Conversely, reduced collagen expression in atrophied muscle tissues of rainbow trout results a decline in shear force [7]. These results collectively suggest a significant association between collagen content and muscle hardness in fish, where higher collagen levels are linked to greater muscle firmness.

In this study, various complementary methods (including Picrosirius red staining, total collagen colorimetric quantification, TEM) confirmed that the YM *T. siluroides* had a lower muscle collagen content than the WT *T. siluroides*. These results collectively suggest that the decrease in collagen content may be the primary structural factor contributing to the reduced muscle hardness in YM *T. siluroides*, consistent with previous observations in vertebrates linking diminished collagen to tissue hardness [45].

### 4.3. The TGF-β/BMP Pathway Mediates Collagen Regulation and Phenotypic Linkages

To explore the molecular mechanisms underlying the differences in collagen reduction and hardness between WT and YM *T. siluroides*, transcriptomic and proteomic analyses were performed. Transcriptomic and proteomic analyses revealed that the TGF-β signaling pathway was a key node associated with differences in muscle hardness between WT and YM *T. siluroides*, with DEGs and DEPs significantly enriched in this pathway. GSEA further confirmed that collagen-related terms and the ECM-receptor interaction pathway were suppressed in YM *T. siluroides*. TGF-β has been shown to promote myoblast differentiation, ECM expression, and facilitate collagen synthesis [46]. This pathway governs skeletal muscle growth by activating SMAD genes to transmit signals and regulate transcription in the nucleus [47]. In humans, activation of the TGF-β signaling pathway is implicated in various muscle-related diseases, including skeletal muscle fibrosis [48]. In mice, induction of this pathway leads to increased type I collagen accumulation [49]. Our results indicate that the reduction in muscle hardness in YM *T. siluroides* might be attributed to the suppression of collagen-related terms and ECM-related pathways, highlighting the significant contribution of the TGF-β signaling pathway.

The downregulation of core TGF-β/BMP pathway molecules (e.g., *BMP2*, *FMOD*, and *SMAD1*) in YM *T. siluroides* correlated with the reduced expression of collagen-related genes (e.g., *COL1A1* and *COL1A2*) and ECM-related genes (e.g., *TNC* and *FN1*), establishing a mechanistic chain that linked pathway activity to muscle structural integrity. *BMP2*, a member of the TGF-β superfamily, regulates collagen by activating intracellular signaling pathways [50]. This activation leads to increased collagen gene transcription, protein synthesis, and extracellular matrix assembly [51]. Within grass carp, SMAD4-dependent TGF-β1 signaling is implicated in the augmentation of type I collagen synthesis [19]. TGF-β and SMAD4 were identified as critical regulatory factors for *COL1A1* and *COL1A2* in vitro studies involving grass carp and the zebrafish ZF4 cell line [18]. In contrast, in *T. siluroides*, BMP2/SMAD1 may act as a key mediator in the TGF-β signaling cascade for type I collagen synthesis, highlighting species-specific differences.

The collagen family comprises more than 20 types of collagen proteins, with type I collagen being the predominant collagen in the intramuscular connective tissue of teleosts [52,53]. Type I collagen consists of α1 and α2 peptide chains encoded by the *COL1A1* and *COL1A2* genes, respectively [6]. The integrated analysis of the transcriptome and proteome in this study further validated *COL1A1a* as a key effector, with consistent downregulation at both the RNA and protein levels. This finding mirrors the findings of grass carp studies in which SMAD4 overexpression significantly upregulated *COL1A1* and *COL1A2*, reinforcing the functional link between TGF-β/BMP activity and collagen deposition [18]. While *COL1A1b* showed divergent RNA-protein expression, this discrepancy may reflect post-transcriptional regulation, a phenomenon that has been documented in teleosts, where gene duplication events often lead to sub-functionalization [54]. Collectively, these findings establish the TGF-β/BMP pathway as a central hub integrating collagen synthesis and ECM stability in *T. siluroides*. Given the close association between collagen, a key component of the extracellular matrix, and muscle firmness, the TGF-β/BMP pathway may be responsible for the reduced muscle hardness in YM *T. siluroides*.

Melanocyte stem cells (McSCs) and melanocytes are pivotal for skin pigmentation [55]. Previous studies have demonstrated that BMP2 promotes melanogenesis by upregulating tyrosinase expression in mature melanocytes [20]. Additionally, accumulating evidence indicates that TGF-β/BMP signaling facilitates melanocyte regeneration [21]. Our study revealed decreased expression of *TYRP1* (a key regulator of melanin synthesis) and *BMP2* in YM *T. siluroides*. Reduced *BMP2* expression may impair melanogenesis by decreasing tyrosinase levels in mature melanocytes; in YM *T. siluroides*, this downregulation likely suppresses TYRP1-mediated melanin biosynthesis and disrupts muscle collagen synthesis, resulting in body colour alterations and differences in muscle hardness. These findings indicate that pathway suppression in YM *T. siluroides* may be involved in the potential association between muscle collagen synthesis (hardness) and melanocyte function (body colour). Such pleiotropy provides a novel potential mechanistic link between body color variation and muscle quality, highlighting the potential coregulation of traits via a single signaling pathway—a phenomenon that is rarely reported in fish but relevant to adaptive trait integration.

The concurrent reductions in TYRP1 and TGF-β/BMP pathway components provide a framework for understanding the potential parallel variation in body colour and muscle hardness in *T. siluroides*, highlighting the BMP2-SMAD1-TYRP1 axis as a valuable target for investigating trait covariation in teleosts.

### 4.4. Limitations and Future Research Directions

The present study offers insights into the potential mechanistic connection between the TGF-β/BMP pathway and simultaneous changes in muscle hardness and body colour in *T. siluroides*, as substantiated by histological, transcriptomic, and proteomic analyses. However, two key points warrant further investigation: first, while pathway components (*BMP2*, *SMAD1*), collagen-related genes (*COL1A1a*, *COL1A1b*), and pigmentation genes (*TYRP1*) show coordinated expression changes, functional evidence for causal relationships remains to be established. Second, the current data are derived primarily from muscle tissue, leaving unanswered whether the BMP2-SMAD1-TYRP1 axis operates similarly in skin (primary pigmentation tissue) or whether paracrine crosstalk between muscle and skin contributes to the observed phenotypic link.

To address these issues, ongoing work includes the establishment of CRISPR-Cas9-edited *T. siluroides* models. In future experiments, these models will be used to perform BMP2 knockdown or SMAD1 overexpression and assess whether such manipulations rescue collagen deposition and body colour phenotypes to confirm causality. Additionally, single-cell RNA sequencing of muscle and skin will clarify whether the pathway acts in overlapping cell types or via paracrine signaling to coordinate pigmentation and muscle traits.

## 5. Conclusions

In summary, this study clarified the differences in muscle quality and their regulatory basis between WT and YM *T. siluroides*. TPA was associated with significantly greater muscle hardness in WT *T. siluroides*, with analyses ruling out myofiber diameter and density as contributing factors. Instead, evidence from multiple methods (Picrosirius red staining, collagen quantification, and TEM) demonstrated that reduced collagen in YM *T. siluroides* was the key structural cause of reduced muscle hardness. Transcriptomic and proteomic analyses highlighted the TGF-β/BMP signaling pathway as a central regulator. The enrichment of DEGs/DEPs in this pathway, combined with the GSEA results showing suppressed collagen-related terms and ECM-receptor interaction pathway, indicated inhibition in YM *T. siluroides*. The downregulation of core molecules (e.g., *BMP2* and *FMOD*) in YM *T. siluroides* reduced the expression of collagen genes (e.g., *COL1A1* and *COL1A2*) and ECM-related genes (e.g., *TNC* and *FN1*), impairing collagen synthesis and ECM stability, which is consistent with the structural changes. Multiomic analyses confirmed the downregulation of *COL1A1a* in YM *T. siluroides*, indicating potential TGF-β/BMP-mediated collagen suppression. Notably, the downregulation of *TYRP1* in YM *T. siluroides* linked attenuation of the TGF-β/BMP pathway to both muscle quality (via collagen/ECM) and body colour (via melanin synthesis), revealing a dual regulatory mechanism that rarely been reported in fish. The present study exclusively uncovers the potential correlational link between the BMP2-SMAD1-TYRP1 axis and the co-variation in body color and muscle hardness in *T. siluroides*. The establishment of their causal relationship necessitates subsequent functional experiments, such as gene knockdown, overexpression, and CRISPR-based gene editing. Our findings may improve the understanding of muscle quality regulation in *T. siluroides* and offer molecular targets for the breeding of varieties with desirable colour and superior muscle quality in aquaculture.

## Figures and Tables

**Table 1:** Muscle texture indicators of WT and YM *T. siluroides*.

Group	Hardness/ (g)	Chewiness/ (g · sec)	Springiness/ (g · sec)	Cohesiveness/ (g · sec)	Gumminess/ (g · sec)	Resilience
WT	824.26 ± 156.35 *	292.04 ± 85.67	0.92 ± 0.07	0.48 ± 0.05	346.48 ± 74.47 *	0.34 ± 0.03 *
YM	558.52 ± 90.66	225.54 ± 68.17	0.91 ± 0.03	0.48 ± 0.06	266.64 ± 57.97	0.29 ± 0.06

* indicates significant difference (*p*  <  0.05).

**Table 2:** Transcriptome sequencing and assembly data of WT and YM *T. siluroides*.

Sample	Raw Data (bp)	Clean Data (bp)	Q20 Rate (%)	Q30 Rate (%)	GC (%)
WT1	5,437,641,300	5,422,137,909	99.28	97.83	49.00
WT2	6,819,118,500	6,801,906,889	99.35	98.00	48.72
WT3	6,110,957,100	6,090,850,604	99.37	98.08	49.35
YM1	5,910,464,400	5,890,548,425	99.36	98.06	48.24
YM2	6,096,620,700	6,071,460,680	99.34	98.01	48.68
YM3	5,579,437,200	5,565,578,952	99.31	97.84	48.31

## Data Availability

The data presented in this study are openly available in the Genome Sequence Archive (GSA) of the China National Center for Bioinformation (CNCB) and PRIDE partner repository, reference number CRA028439 and PXD066809.

## Appendix A

**Table A1:** Gene-specific primers for qRT-PCR.

Gene	Sequence (5′ to 3′)	Product (bp)
SMAD2-F	GTCCTCCATCTTGCCTTTCAC	178
SMAD2-R	GTCCAGTTGACCCGTTTTCTT	
DDIT4-F	CAAGCCTCGTGTCGGAAGA	213
DDIT4-R	TGGGGTCAAAGAACAGGTCA	
DCTN1-F	GAACCTAAAGGCAAGAACGATG	145
DCTN1-R	CGGCTTCAGGGGTTTCAG	
TGF-β1a-F	CATTGGAAATAAAACCCGCTAC	126
TGF-β1a-R	GCTTGATTCTCTTCTGACTTCTGC	
BMP2-F	TCAGAGACCCCTACTGGTTACTTAC	190
BMP2-R	GTTGCGTTTCACCCGTCTT	
SMAD3a-F	GCAGGTATCTCATAGGAAAGGACT	106
SMAD3a-R	CTCACAGTGTTCAATGGCTCTAAG	
SMAD4-F	GTTCTGCCAGTATGCCTTTGAC	228
SMAD4-R	GCCACAGGTCTGACAGGAGG	
COL1A1a-F	GTTCAGCTTTGTGGATATTCGGT	226
COL1A1a-R	GTTGGGGCAGTCGGTCGT	
COL1A1b-F	GCAGCGGTCCTTGTGGTG	194
COL1A1b-R	ATTTCTGGATTGGCACAGTCTG	
COL2A1-F	ACCAAGCGGCAGAAGGAA	150
COL2A1-R	CGGGGTGACAGAGTTTGAGAT	
HBB-F	AGGTGGCAGCTCATGGTAGAA	202
HBB-R	TCAGCATTGAAAACTGTGGGTC	
CTNNB1-F	GGCAACCCAGAGGAAGAGG	161
CTNNB1-R	TCCAAAGTCTCGGGGAACAT	
β-actin-F	GCTGAGAGAGAAATTGTCCGTG	209
β-actin-R	CCGCAAGACTCCATACCCA	
